# Efficacy of a Novel Augmented Reality Navigation System Using 3D Computer Graphic Modeling in Endoscopic Transsphenoidal Surgery for Sellar and Parasellar Tumors

**DOI:** 10.3390/cancers15072148

**Published:** 2023-04-04

**Authors:** Yoshiaki Goto, Ai Kawaguchi, Yuki Inoue, Yuki Nakamura, Yuta Oyama, Arisa Tomioka, Fumi Higuchi, Takeshi Uno, Masaaki Shojima, Taichi Kin, Masahiro Shin

**Affiliations:** 1 Department of Neurosurgery, University of Teikyo Hospital, 2-11-1 Kaga, Itabashi-ku, Tokyo 179-8606, Japan; 2 Department of Neurosurgery, University of Tokyo Hospital, 7-3-1 Hongo, Bunkyo-ku, Tokyo 133-8655, Japan

**Keywords:** augmented reality, navigation system, endonasal transsphenoidal surgery, parasellar tumors, neuroendoscopy

## Abstract

**Simple Summary:**

In endoscopic transsphenoidal skull base surgery, it is often difficult to accurately determine the location of a tumor and its surroundings on imaging because the lesion remarkably displaces the geography of normal anatomic structures. We aimed to create a novel augmented reality (AR) navigation system that could compensate for this displacement effect. We created a precise three-dimensional computer graphic (3DCG) model that we superimposed on a visual image of the actual surgical field and displayed on a video monitor during endoscopic transsphenoidal surgery. Surgeons evaluated its efficacy using a five-point scale in 15 consecutive patients with sellar and parasellar tumors. The average score overall was 4.7 (95% confidence interval: 4.58–4.82), and the AR navigation system was considered as useful as or more useful than conventional navigation in certain patients. This system has the advantage of facilitating an immediate 3D understanding of the lesion and surrounding structures.

**Abstract:**

In endoscopic transsphenoidal skull base surgery, knowledge of tumor location on imaging and the anatomic structures is required simultaneously. However, it is often difficult to accurately reconstruct the endoscopic vision of the surgical field from the pre-surgical radiographic images because the lesion remarkably displaces the geography of normal anatomic structures. We created a precise three-dimensional computer graphic model from preoperative radiographic data that was then superimposed on a visual image of the actual surgical field and displayed on a video monitor during endoscopic transsphenoidal surgery. We evaluated the efficacy of this augmented reality (AR) navigation system in 15 consecutive patients with sellar and parasellar tumors. The average score overall was 4.7 [95% confidence interval: 4.58–4.82], which indicates that the AR navigation system was as useful as or more useful than conventional navigation in certain patients. In two patients, AR navigation was assessed as less useful than conventional navigation because perception of the depth of the lesion was more difficult. The developed system was more useful than conventional navigation for facilitating an immediate three-dimensional understanding of the lesion and surrounding structures.

## 1. Introduction

For more than two decades, endoscopic transsphenoidal surgery (ETS) has been widely accepted as a safe approach for pituitary tumors, and its indication has recently been expanded for tumors in various skull base regions [1,2,3,4,5]. During ETS for pituitary lesions, we identify anatomical landmarks in the sphenoid sinus such as the sella turcica, clival recess, optic canals, and carotid prominence, and the sellar floor can be safely opened to remove the tumor inside without special guidance from a neuronavigation system. When this surgical approach is expanded to tumors located in the skull base, comprehension of the surrounding anatomy becomes essential [6,7,8,9,10,11]. For endoscopic transsphenoidal skull base surgery, lesions as well as anatomic structures including cranial nerves, important cerebral structures, and critical vascular structures from the limited landmarks in the sphenoid sinus must be located simultaneously on imaging. However, it is often difficult to accurately reconstruct the endoscopic vision of the surgical field from pre-operative radiographic imaging because the lesions remarkably displace the geography of the normal anatomic structures [12,13].

To compensate for this situation, neuronavigation is used for complex neurosurgical procedures [14,15,16,17]. It realizes accuracy to less than a few millimeters to indicate the real-time location on preoperative computed tomography (CT) or magnetic resonance images (MRI), and vision is compared between the surgical field and the two-dimensional (2D) radiographic images [14,18]. Furthermore, neuronavigation occasionally has a margin of error that causes difficulties in immediate decision making. To resolve the ambiguity associated with the discrepancy between the visual image of the actual surgical field and the 2D preoperative radiographic images, a three-dimensional computer graphic (3DCG) model that uses the patient’s radiographic images has been developed and is becoming accepted for clinical use [19,20,21,22,23,24,25,26,27,28,29,30].

Several studies have suggested the possible application of this technology for clinical use in endoscopic skull base surgery [23,27,28,29,30]. There are two cadaveric [23,27] and four clinical studies [28,29,30] in which the conventional navigation system for topographic adjustment was used, and three-dimensional images created from the preoperative images represent only the outline of the anatomic structures. However, to our knowledge, visualization of the comprehensive neuroanatomy to the level of detail required for the meticulous surgical procedure of skull base surgery is currently only in the development stage. In addition, studies on the development of a self-sustaining AR navigation system with a novel topographic adjustment method are scarce.

In this study, we developed a unique AR navigation system, which applied detailed three-dimensional computer graphics (3DCG) created from the preoperative image and using the intuitive real-time adjustment method. The aim of this study was to evaluate the efficacy of this augmented reality (AR) neuronavigation system during ETS for parasellar tumors, based on the 3DCG model created from the preoperative radiographic data.

## 2. Materials and Methods

### 2.1. Patients

We evaluated the efficacy of AR navigation using a 3DCG model in 15 consecutive patients who underwent ETS for sellar and parasellar tumors. Details of the patient characteristics are summarized in Table 1. Our ethics review board approved the study, and written informed consent was obtained from all patients. During surgery, the AR navigation system was used together with a conventional navigation system (SteathStation S8^TM^, Medtronic, Minneapolis, MN, USA), in which the target point was indicated on 2D radiographic images.

### 2.2. DCG Model Creation from Clinical Radiographic Images

#### 2.2.1. Radiographic Image Acquisition

CT and MRI with gadolinium enhancement were acquired before surgery in all patients and were used to create the 3DCG VR model. CT was performed with a 320-slice CT scanner (Aquilion ONE TSX-306A; Canon Medical Systems, Tokyo, Japan) using the following parameters: collimation, 0.5 mm; tube voltage, 120 kVp; tube current (volume exposure control), 100–500 mA; rotation time, 0.75 s; reconstruction section width, 0.5 mm; reconstruction interval, 0.5 mm; number of slices, 640; and voxel size, 0.43 mm × 0.43 mm × 0.5 mm. MRI was performed with a Signa 3.0-T system (GE Healthcare, Milwaukee, WI, USA). Imaging parameters for gadolinium-enhanced T1 weighted images were as follows: repetition time, 7.0 ms; echo time, 3.2 ms; slice thickness, 0.7 to 1.4 mm; field of view, 22 cm; matrix size, 352 × 256; flip angle, 13 degrees; voxel size, 0.63 mm × 0.85 mm × 0.7 mm; and timing, 4 min after contrast injection. The imaging parameters for Fast Imaging Employing Steady-State Acquisition (FIESTA) were as follows: repetition time, 5.6 ms; echo time, 2.7 ms; slice thickness, 0.4 to 0.8 mm; field of view, 20 cm; matrix size, 320 × 320; flip angle, 50 degrees; voxel size, 0.63 mm × 0.63 mm × 0.4 mm; and timing, just after contrast injection. Imaging parameters for 3D time-of-flight magnetic resonance angiography (TOFMRA) were as follows: repetition time, 26 ms; echo time, 2.7 ms; slice thickness, 0.5 to 1.0 mm; field of view, 20 cm; matrix size, 384 mm × 224; flip angle, 20 degrees; and voxel size, 0.63 mm × 0.89 mm × 0.5 mm.

#### 2.2.2. Image Processing

The reconstruction method for creating 3DCG models from clinical 2D images has been reported previously [27,28,29]. Briefly, CT and MRI data in digital imaging and communications in medicine (DICOM) format were processed using 3D simulation software for medical images (GRID, Kompath Inc. Tokyo, Japan). These datasets were automatically registered using the normalized mutual information method. Each target tissue and organ was created independently as a 3DCG model using optimally visualized thresholds. To make fusion models, all modalities and sequences were aligned by performing registration of the two types of image data in order, and their tissue boundaries were checked against the 2D original image. Finally, all surface-rendered models were reconstructed as polygonal models. The resulting 3D reconstruction model was transferred to a portable tablet computer (iPad Pro 12.9-inch 6th generation, Apple Inc., Sunnyvale, CA, USA) prior to each patient’s surgery and was displayed and controlled with an 3D viewer application (LIVRET, Kompath Inc., Tokyo, Japan).

#### 2.2.3. Creation of the Fused Image

We performed endoscopic skull base surgery while facing images of the surgical field displayed on two 43-inch monitors, of which the second monitor was used for AR navigation. The optical images obtained by a 4K rigid neuroendoscope (KTH121 IMAGE1 S™ 4U Rubina^TM^, KARL STORZ Endoscopy Japan K.K., Tokyo, Japan) were displayed on the main monitor and also output simultaneously to a video mixer (Roland V-02HD MKII, Roland Corporation, Shizuoka, Japan). The video data of the surgical field and the 3DCG image in the portable tablet computer were separately input into the video mixer, and those 2 images were superimposed on the second monitor for preparation of the topographic adjustment.

#### 2.2.4. Creation of the AR Navigation Image on the Second Monitor during Endoscopic Surgery

**Figure 1:**
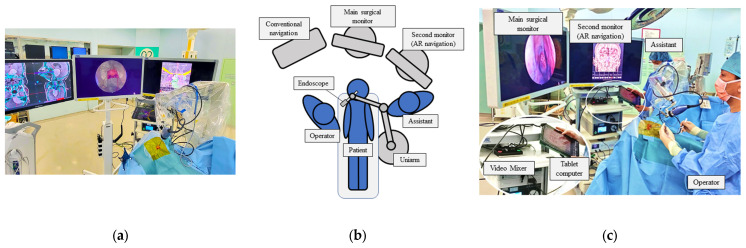
Setup of the operation room with AR navigation (**a**). We used double monitors, with the middle one for surgical procedure and the left one for the AR navigation, to present a composite surgical view (**b**,**c**).

The patient was placed supine with the head raised 15 degrees. The head was fixed with a Mayfield 3-point head holder, rotated slightly toward the operator. We used double monitors during the surgery, one for surgery and one for AR navigation, and the navigation system was controlled by the assistant (Figure 1a–c).

**Figure 2:**
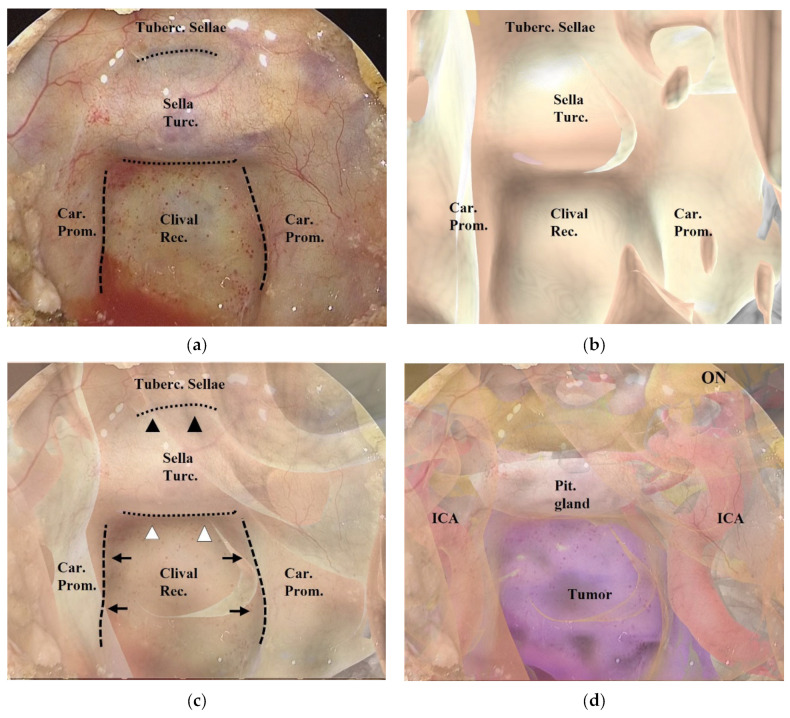
Procedure for superimposing the 3DCG model on the neuroendoscopic image. (**a**) Endoscopic image in the sphenoid sinus. The dashed lines indicate the inner lines of the bilateral carotid prominences, and the dotted lines indicate the inferior line of the tuberculum sellae and the sellar floor. (**b**) 3DCG model in the sphenoid sinus. (**c**) Fusion image created by superimposing the 3DCG model (**b**) on the endoscopic image (**a**). The inner lines of the bilateral carotid prominences (black arrows) were aligned with the dashed lines, and the 3DCG was tilted to align the sellar floor (white arrowheads) and the tuberculum sellae (black arrowheads) with the dotted lines. (**d**) Fusion image with intracranial structures displayed in the 3DCG model. Tuberc., tuberculum; Rec., recess; Turc., turcica; Car., carotid; Prom., prominence; Pit., pituitary; ON, optic nerve; ICA, intracranial artery.

After wide anterior sphenoidotomy, characteristic structures in the sphenoid sinus, such as the sella turcica, clival recess, optic prominence, optico-carotid recess, and carotid prominence, were exposed (Figure 2a). The assistant adjusted the position of the 3DCG model on the tablet to enable these structures to be identified on the 3DCG model (Figure 2b). After setting these landmark structures, the 3DCG image was then superimposed on the vision of the surgical field.

**Figure 3:**
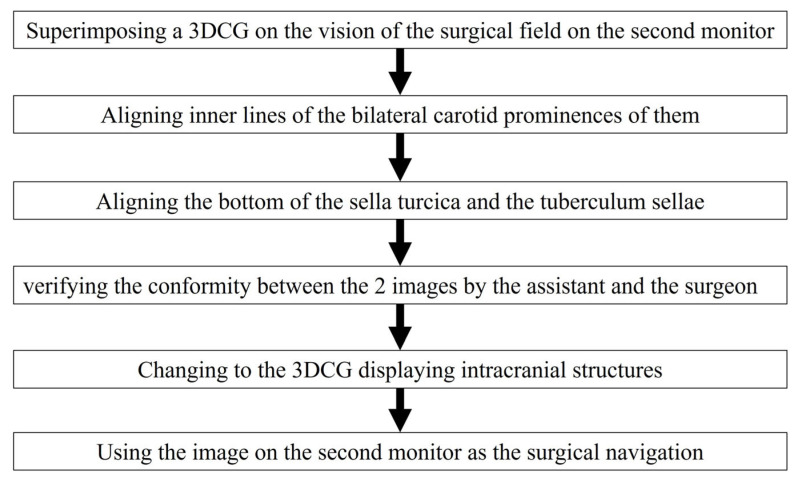
Flow chart for aligning the 3DCG with the vision of the surgical field.

Adjustment of the image on the vision of the surgical field was controlled by the assistant (Figure 2a–c and Figure 3). Initially, we noted the alignment of the carotid prominences (CP), and they were adjusted by shifting and then scaling the 3D image on the second monitor. Additionally, a slight adjustment in rotation of the image was done to sufficiently match the bottom lines of the sella turcica and the tuberculum sellae (Video S1). Finally, the assistant and the surgeon verified the conformity between the 2 video images and agreed to use it as the surgical navigation (Figure 2d). After tumor exposure, dissection from the surrounding anatomy was performed. The dural defect was closed with fascia, which was placed in the multilayered fashion and was sutured with the absorbable polydioxanone suture (STRATAFIX; Johnson & Johnson K.K. Tokyo, Japan).

### 2.3. Evaluation of the AR Navigation System in ETS

We used an original five-point scale to evaluate the efficacy of the AR navigation system (Table 2). The two neurosurgeons who performed the endoscopic skull base surgery and the three senior residents who participated in ETS evaluated the efficacy of the AR navigation system after the surgery.

## 3. Results

**Figure 4:**
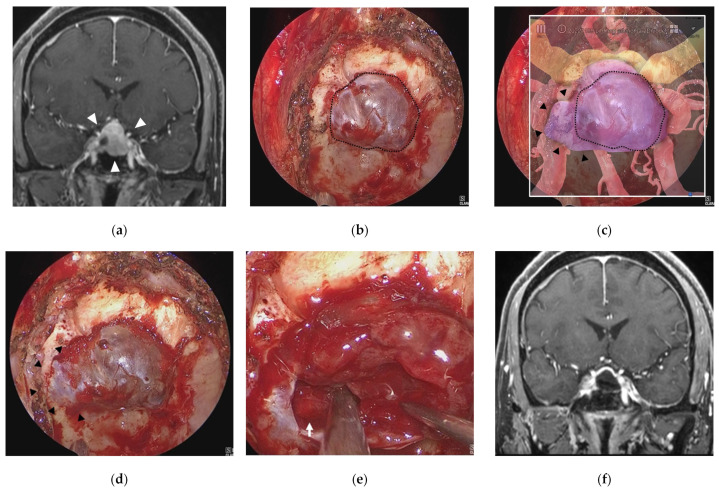
Illustrative case 1. (**a**) Coronal section of preoperative gadolinium enhanced T1 weighted image shows a sellar tumor with suprasellar extension (white arrowheads). (**b**) Endoscopic view of the sphenoid sinus after removal of bone around the sella turcica (dotted line). (**c**) Fusion image of the endoscopic view (**b**) and the semi-transparent 3DCG model shows extension of the pituitary tumor into the right cavernous sinus (black arrowheads) beyond the bone removal line. (**d**) Endoscopic view after additional bone removal (black arrowheads). (**e**) The right ICA (white arrow) is exposed after resection of the pituitary tumor around the cavernous sinus. (**f**) Coronal section of postoperative gadolinium-enhanced T1 weighted image shows total removal of the tumor. ICA = intracranial artery.

**Figure 5:**
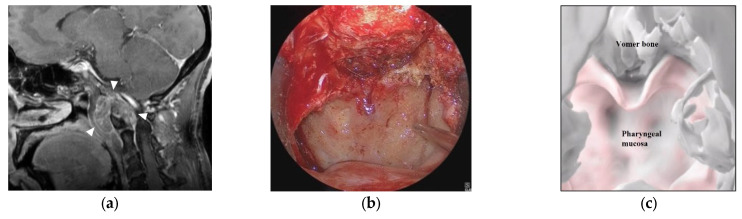

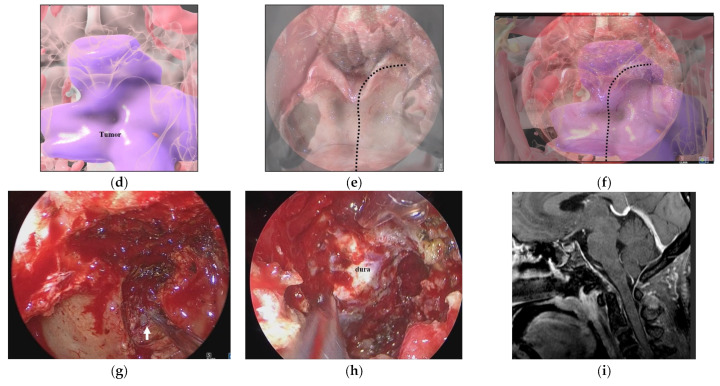
Illustrative case 2. (**a**) Sagittal section of preoperative gadolinium enhanced T1 weighted image shows a tumor around the craniovertebral junction (white arrowheads). (**b**) Neuroendoscopic image in the nasal cavity shows the pharyngeal mucosa and the inferior edge of the vomer bone. (**c**) 3DCG of the nasopharyngeal mucosa and skull shows the pharyngeal mucosa and vomer bone. (**d**) 3DCG with semi-transparent pharyngeal mucosa shows the tumor under the mucosa. (**e**) Fusion image of the neuroendoscopic image (**b**) and the 3DCG of the nasopharyngeal mucosa (**c**). Dotted line indicates the curved cutting line of the mucosa. (**f**) Fusion image of the neuroendoscopic image (**b**) and the 3DCG with semi-transparent mucosa (**d**). (**g**) Neuroendoscopic image after cutting the nasopharyngeal mucosa reveals the tumor (white arrow). (**h**) Neuroendoscopic image after tumor removal shows the dura. (**i**) Coronal section of postoperative gadolinium-enhanced T1 weighted image shows total removal of the tumor.

**Figure 6:**
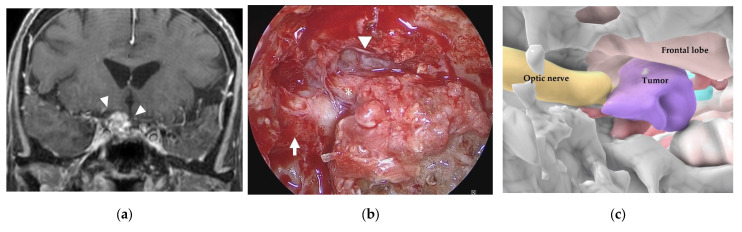

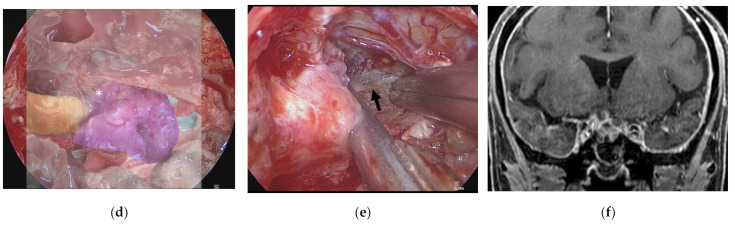
Illustrative case 3. (**a**) Coronal section of preoperative gadolinium enhanced T1 weighted image shows a tumor around the right optic nerve (white arrowheads). (**b**) Neuroendoscopic image in the sphenoid sinus after cutting the thick mucosa shows the right optic sheath (white arrow), right frontal lobe (white arrowhead), and a tumor-like structure (white asterisk). (**c**) 3DCG shows the location of the tumor medial to the right optic nerve and inferior to the frontal lobe. (**d**) Fusion image shows that the tumor-like structure is coincident with the tumor observed in the 3DCG (white asterisk). (**e**) The right optic nerve (black arrow) is exposed after removal of the tumor. (**f**) Coronal section of postoperative gadolinium-enhanced T1 weighted image shows subtotal removal of the tumor.

During ETS, AR navigation was used systematically on two occasions: before drilling the skull base bony structures (Figure 2 and Figure 4) and after drilling and before opening the mucosa or dura mater, to confirm the anatomic structures hidden behind and also that the area of mucosal or dural opening was adequate (Figure 5). In addition, it was used in certain circumstances, such as after exposure of the tumor outlines (Figure 6). In each of the 15 patients, a 3DCG model was successfully created from the preoperative radiographic images, and AR navigation was used in their ETS.

Table 3 and Table S1 show the results of assessment using the five-point scale for each patient. The neurosurgeons and residents evaluated the efficacy of the AR navigation as score 5 in 5/15 cases (Cases 6, 9, 10, 11, and 15) and as ≥4 in 13/15 patients. The average score overall was 4.7 (95% confidence interval (CI): 4.58–4.82), which indicates that AR navigation was more useful than conventional navigation for certain patients. A score of 1 or 2 was not recorded in any patient, and in no case did AR navigation misguide the neurosurgeon or resident during ETS. In assessment by the three residents, two cases (Cases 5 and 7) were scored as 3 (AR navigation was not as useful as conventional navigation), by only one resident in each case. In these cases, the main complaint was that perception of depth (antero–posterior length) of the lesion was more difficult with AR navigation. Thus, there might be individual differences among surgeons regarding recognition of the virtual image as the ‘real’ situation, depending on their previous experience with similar cases in ETS. Patient outcomes are listed in Table 4.

### 3.1. Illustrative Case 1 (Case 4)

A 58-year-old man underwent ETS for a growing pituitary neuroendocrine tumor (PitNET). Preoperative MR imaging showed an intrasellar tumor with suprasellar extension (Figure 4a). After removal of bone of the sella turcica (Figure 4b) in ETS for a PitNET, AR navigation showed that the tumor extended into the right cavernous sinus and displaced the ICA laterally (Figure 4c). After additional bone removal from the right cavernous sinus (Figure 4d), the dura mater was incised, and the tumor was sufficiently exposed. The tumor was totally removed, and the cavernous segment of the right ICA was exposed (Figure 4e). The tumor was totally resected, and the patient had a good postoperative course (Figure 4f).

### 3.2. Illustrative Case 2 (Case 9)

A 50-year-old woman had a hypoglossal nerve palsy due to a chordoma and underwent ETS. Preoperative MR imaging showed a tumor at the craniovertebral junction (Figure 5a). A skull base chordoma involving the craniovertebral junction was not evident from the nasal cavity (Figure 5b), and the extent of tumor invasion was not apparent on endoscopic vision. On AR navigation, we created models of the nasopharyngeal mucosa (Figure 5c) and the tumor (Figure 5d), which were superimposed on the surgical field during ETS (Figure 5e,f) and clearly disclosed the extent of tumor invasion (Figure 5f). A curved mucosal incision was made to identify the tumor behind the mucosa (Figure 5g). The tumor was dissected from the submucosal tissue and removed. The tumor was completely resected under the vision of an angled scope (Figure 5h). The tumor was totally resected (Figure 5i), and the patient’s hypoglossal nerve palsy improved gradually.

### 3.3. Illustrative Case 3 (Case 10)

A 75-year-old woman had right visual disturbance due to recurrent meningioma. Preoperative MR imaging showed a tumor around the right optic nerve (Figure 6a). In a patient with recurrent tuberculum sellae meningioma, endoscopic images in the sphenoid sinus showed thick mucosa. After cutting the mucosa, a tumor-like structure was observed inferior to the right frontal lobe (white arrowhead) and medial to the right optic sheath (Figure 6b). There was a part of the tumor that was difficult to distinguish from the displaced optic nerve from the color or the consistency. However, the AR navigation indicated it as a “tumor” (Figure 6c,d), and we could resect this part with confidence (Figure 6e). The tumor was subtotally resected (Figure 6f), and the patient’s visual disturbance gradually improved.

## 4. Discussion

In this report, we present the preliminary results of our novel AR navigation system in ETS. The average score overall indicated that the surgeons considered AR navigation to be more useful than conventional navigation for certain patients. On the other hand, in two cases it was evaluated that AR navigation was not as useful as conventional navigation, though by only one resident in each case. The main complaint involved perception of depth; there might be individual differences among surgeons regarding recognition of the virtual image as the ‘real’ situation, depending on their previous experience with similar cases in ETS.

Compared to the conventional navigation system that uses infrared light or magnetic field guidance for topographic adjustment [31], our AR navigation system recognizes the gross landmarks of the anatomic structures and superimposes them on the created images, which has several advantages. First, in this AR navigation system, the created image is identical to the image of the surgical field [32,33,34,35]. Thus, it is easy to superimpose the image on the surgical anatomy, and we can comprehend the anatomy of the surgical field more intuitively, which is a major advantage of our system.

In the conventional navigation system, a single point is indicated on the radiographic image. Thus, we must verify the anatomical location from the radiographic images presented on the monitor in the multiple sections and simultaneously estimate the margin of error, including the shifts in the x, y, and z directions. Even if there is an apparent displacement between the actual anatomy and the indicated point on the radiographic image, there is no automatic method to detect and correct the discrepancy. Therefore, to successfully use the conventional navigation system, we must bear in mind the possibility of a minor shift and readily correct it based on comparison of the anatomical topography in the surgical field and the 2D radiographic images on the navigation monitor. Our AR navigation system embraces those human abilities to correct the technological error. The accuracy and efficacy of our AR navigation system largely rely on the subjective involvement of humans, much more so than the conventional navigation system. Because our AR navigation applies a high-quality 3D image, the sensing capability of displacement in the navigation is much higher than that of conventional navigation, which can be easily corrected and may reduce the margin of error to a minimum.

Although the anatomic structures in the sphenoid sinus are generally identical and easily identifiable during ETS, the AR navigation system is particularly useful for revealing the anatomic structures hidden behind the bony structure or the tumor in the surgical field. We often hesitate to resect tumors with a similar appearance or color to the surrounding normal anatomy, even if it has been recognized by conventional navigation as a “tumor”, considering the possible margin of error. In contrast, our AR navigation system enables comprehensive recognition of the target lesion in relation to the surrounding landmark anatomies, which enables direct assistance for surgical decision-making.

Our AR navigation system may be more useful in young neurosurgeons who do not have significant experience with endoscopic transsphenoidal surgery. In the case of the sellar tumor (presented in the illustrative case 1), for the neurosurgeons who have certain experience with endoscopic transsphenoidal surgery, the tumor extension in the sella and position of the displaced internal carotid artery are readily recognized in the surgical field by reference to the 2D MRI. The AR navigation is used only for confirmation of their outlooks. In contrast, young neurosurgeons are less confident in identification of such “distorted” anatomies, and the AR navigation is useful for them to verify location of these important anatomies. Simultaneously, for young neurosurgeons, the AR navigation is also useful for surgical education to reconstruct the image of the surgical field from the preoperative 2D MRI in their mind and retrospectively evaluate the accuracy of their outlook of the surgical field. Furthermore, in ETS for complex skull base lesions, our system is useful, regardless of the surgeon’s experience. In the illustrative case 2, the AR navigation successfully disclosed the area of tumor extension in the retropharyngeal space, contributing sufficient exposure of the tumor. In the illustrative case 3, it was difficult to distinguish the tumor from the displaced optic nerve using the color or the consistency. Because we could clearly distinguish the optic nerve from the recurrent tumor on MRI, the AR navigation created with 3DCG from the preoperative MRI clearly indicated it as a “tumor”, and we could successfully resect the tumor.

On the other hand, as limitations, it is very difficult to objectively evaluate the margin of error, which is one of the major disadvantages associated with our AR navigation. Additionally, to accurately superimpose the 3D image on the surgical field, we always require identifying the multiple anatomic landmarks. Thus, when the tumor extensively invades into the skull base regions, destroying or harboring the characteristic anatomical landmarks, it will be difficult to accurately superimpose the 3DCG, hampering its use in surgical navigation. The present AR navigation system has some issues that must be addressed. It takes more than one hour to create the 3DCG models with precision, depending on tumor location and the number of critical structures involved in the tumor [32,33]. Thus, our AR navigation system requires a certain preparation time, and it is difficult to incorporate any new changes in the radiographic images immediately before surgery. In addition, in case of tumor recurrence, the anatomical landmarks may already have been destroyed, which makes AR imaging difficult. We are currently developing a system for automatic alignment of the AR navigation to solve such issues. Technological progress in this area will further facilitate the registration of the image on the surgical field and enhance the efficacy of our AR navigation system.

## 5. Conclusions

In this study, we report the preliminary results of our novel AR navigation system during ETS for sellar and parasellar tumors. The resulting 3DCG images were identical to structures in the surgical field. Surgeons considered the present system more useful than the conventional navigation system for facilitating an immediate 3D understanding of the lesion and surrounding structures. Our AR navigation system facilitates intuitive comprehension of the skull base anatomy during ETS and helps not only young neurosurgeons in ETS for basic sellar lesions but also experienced neurosurgeons in ETS for complex skull base lesions. While further refinement of the image fusion techniques is essential, the preliminary results of this study suggest efficacy of our AR navigation in ETS for pituitary and parasellar tumors.

## Figures and Tables

**Table 1:** Clinical characteristics of patients.

Case	Age (y)	Sex	Diagnosis	Approach
1	47	male	Petroclival meningioma	Transsphenoidal-transclival
2	58	male	NF-PitNET	Transsphenoidal
3	82	male	Clival chordoma	Transsphenoidal-transclival
4	58	male	NF-PitNET	Transsphenoidal
5	39	male	Skull base chondrosarcoma	Transsphenoidal-transmaxillary
6	52	female	Intraorbital cavernous malformation	Transsphenoidal-transethmoidal
7	16	male	Craniopharyngioma	Transsphenoidal
8	57	female	PitNET	Transsphenoidal
9	50	female	Craniovertebral junction chordoma	Transsphenoidal-transclival
10	75	female	Tuberculum sellae meningioma	Transsphenoidal
11	58	male	Somatotroph-PitNET	Transsphenoidal
12	60	female	NF-PitNET	Transsphenoidal
13	55	male	NF-PitNET	Transsphenoidal
14	74	female	NF-PitNET	Transsphenoidal
15	56	female	Petroclival meningioma	Transsphenoidal-transclival

NF: non-functional, PitNET: pituitary neuroendocrine tumor.

**Table 2:** Scale for assessment of AR navigation.

Result of Assessment	Score
Misleading, confusing	1
Not misleading but not useful	2
Useful but less so than conventional neuronavigation	3
As useful as conventional neuronavigation	4
More useful than conventional neuronavigation	5

**Table 3:** Results of assessment of AR navigation.

Case	Diagnosis	Surgeon 1	Surgeon 2	Resident 1	Resident 2	Resident 3	95% CI
1	petroclival meningioma	5	5	5	4	5	4.4–5.2
2	PitNET	4	4	5	4	5	3.91–4.89
3	clival chordoma	4	4	5	4	5	3.91–4.89
4	PitNET	5	5	5	5	4	4.4–5.2
5	skull base chondrosarcoma	4	5	3	5	5	3.8–5.4
6	intraorbital cavernous malformation	5	5	5	5	5	5
7	craniopharyngioma	5	4	5	3	5	3.8–5.4
8	PitNET	4	4	5	5	4	4.11–5.09
9	craniovertebral junction chordoma	5	5	5	5	5	5
10	tuberculum sellae meningioma	5	5	5	5	5	5
11	PitNET	5	5	5	5	5	5
12	PitNET	4	4	4	5	5	4.11–5.09
13	PitNET	4	4	4	4	4	4
14	PitNET	4	5	5	5	5	4.4–5.2
15	petroclival meningioma	5	5	5	5	5	5

NF: non-functional, PitNET: pituitary neuroendocrine tumor, CI: confidence interval.

**Table 4:** Patient outcomes.

Case	Age	Sex	Diagnosis	Symptoms at ETS	Tumor Volumes (cm^3^)	Level of Resection	Symptoms after ETS	Postoperative Complications
1	47	M	Petroclival Meningioma	hearing deterioration	26.25	NTR	improved	none
2	58	M	PitNET	oculomotor nerve and abducens nerve palsy	1.76	GTR	improved	none
3	82	M	clival chordoma	abducens nerve palsy	12.90	GTR	improved	none
4	58	M	PitNET	None	4.35	GTR	none	none
5	39	M	skull base chondrosarcoma	unilateral temporal hemianopsia	1.35	GTR	no change	none
6	52	F	intraorbital cavernous malformation	impaired vision, oculomotor disorder	4.37	GTR	improved	none
7	16	M	Craniopharyngioma	headache, vertigo (hydrocephalus)	6.78	NTR	improved	none
8	57	F	PitNET	bitemporal hemianopsia	3.70	GTR	improved	none
9	50	F	craniovertebral junction chordoma	hypoglossal nerve palsy	23.23	GTR	no change	none
10	75	F	tuberculum sellae meningioma	impaired vision, unilateral temoral hemianopsia	1.94	STR	improved	none
11	58	M	PitNET	none	2.31	GTR	none	none
12	60	F	PitNET	homonymous hemianopsia	6.35	NTR	improved	none
13	55	M	PitNET	bitemporal hemianopsia	5.25	GTR	improved	none
14	74	F	PitNET	Bitemporal hemianopsia	5.31	GTR	improved	none
15	56	F	Petroclival Meningioma	vertigo, tinnitus	17.76	STR	improved	Transient abducens nerve palsy

M: male, F: female, PitNET: pituitary neuroendocrine tumor, GTR: gross total resection, NTR: near total resection, STR: subtotal resection.

## Data Availability

All data are contained within the Supplementary Materials (Supplementary Table S1).

## Supplementary Materials

The following supporting information can be downloaded at: https://www.mdpi.com/article/10.3390/cancers15072148/s1, Table S1: Five-point scale evaluation of the augmented reality. Video S1: Procedure video for superimposing the 3DCG on the endoscopic image.
